# Phenomic technologies in drug discovery of neglected diseases

**Published:** 2011-01-10

**Authors:** Ulf Nehrbass

**Affiliations:** 1 Institut Pasteur-Korea, Gyeonggi-do, Korea

## 

Leishmaniasis, Chagas Disease and Dengue are examples of neglected infectious disease that, together with Malaria, are responsible for over 1 million deaths each year. In all cases, new treatments are badly needed. Malaria treatment, although available, is becoming problematic due to worldwide spread of parasite resistance to drugs. For Leishmaniasis, drugs are often ineffective due to the pathogens’ resistance, and in some cases the drugs themselves are potentially deadly for patients. For Chagas, available treatment is often ineffective past the acute phase of the disease, and for Dengue there is no current treatment available at all.

IP-Korea develops Phenomic Screen™, image-based high-content/high-throughput screening assays to find new drugs against Leishmaniasis. Using a whole-cell-based approach, we designed assays that allow selection of compounds that have potent activity against pathogens and are not toxic for the human cells. These cellular assays were adapted to high-throughput for automated image acquisition. Using this technology we have screened 200,000 drugs that impair the growth of the most deadly species causing visceral Leishmaniasis, inside their natural host cell, the human macrophages.

We have also developed a phenotypic screen for Chagas disease and screened 150,000 compounds, in collaboration with Pfizer and the Drugs for Neglected Diseases *initiative* (DND*i*), for their ability to inhibit *Trypanosoma cruzi*, the parasite causing Chagas, inside human host cells. Also, we established visual screening procedure to screen human genome in high content imaging mode within 8h. Genome-wide RNAi screening to identify human host proteins that are required in Chagas disease infection has been finished. The identification of these factors provides potential new targets for anti-parasitic therapies.

Dengue is still in the early stage but is progressing towards the development of a cell-based high-throughput screening assay using different virus serotypes and clinical isolates.

For malaria, we are developing HCS/HTS assays that target the invasion of human red blood cells by the deadliest of all malaria parasites, *P. falciparum*. We have screened 80,000 compounds for their antimalarial activity, and the selected hits will be re-tested for their ability to interfere with the invasion pathway using an image based high-content assay. In parallel, we are developing a phenotypic assay to screen for drugs that interfere with *P. falciparum* infected red blood cells cytoadhesion, one the main pathogenic mechanism of this parasite*.*

